# Synthesis, Structural, Optical, and Electrical Characterization of Biochitosan/Na_0.5_Bi_0.5_TiO_3_ Composite Thin-Film Materials

**DOI:** 10.3390/mi14101841

**Published:** 2023-09-27

**Authors:** Jacem Zidani, Khaoula Hassine, Moneim Zannen, Andreas Zeinert, Antonio Da Costa, Anthony Ferri, Jamal Belhadi, Mustapha Majdoub, Mimoun El Marssi, Abdelilah Lahmar

**Affiliations:** 1 Laboratoire de Physique de la Matière Condensée (LPMC), Université de Picardie Jules Verne, 33 rue Saint-Leu, 80039 Amiens, CEDEX 1, France; jacem.zidani@etud.u-picardie.fr (J.Z.); mimoun.elmarssi@u-picardie.fr (M.E.M.); 2 Laboratory of Interfaces and Advanced Materials (LIMA), Faculty of Sciences of Monastir, University of Monastir, Bd. of the Environment, Monastir 5019, Tunisia; hassinekhaoula1@gmail.com (K.H.); moneimchimie2006@yahoo.fr (M.Z.); mustaphamajdoub@gmail.com (M.M.); 3 University of Artois, CNRS, UMR 8181—UCCS—Unité de Catalyse et Chimie du Solide, 62300 Lens, France; antonio.dacostafereira@univ-artois.fr (A.D.C.); anthony.ferri@univ-artois.fr (A.F.)

**Keywords:** composite films, chitosan, NBTO, dielectric properties, local piezoelectric response, optical properties

## Abstract

The purpose of this research work was to synthesis bioderived nanocomposite films by incorporating Na_0.5_Bi_0.5_TiO_3_ (NBTO) nanoparticles into a chitosan matrix. The NBTO nanoparticles were synthesized using a traditional solid-state technique. Then, through a solution-casting approach, flexible composite films were fabricated using chitosan polymer. The study presents a range of compelling findings. For structural and morphological insights, scanning electron microscopy (SEM) reveals a fascinating morphology where NBTO nanoparticles are uniformly dispersed and interlocked with other particles, forming interconnected grains with significant interspaces within the chitosan matrix. For the optical properties, the spectral response within the 300–800 nm range is primarily governed by light scattering attributed to NBTO particles with diameter sizes ranging from 100 to 400 nm, as well as the distinctive bandgap exhibited by the NBTO phase. The investigation of dielectric properties demonstrates that composite films exhibit markedly higher dielectric values in comparison to pure chitosan films. It is noteworthy that an increase in the NBTO content results in a corresponding increase in dielectric values, enhancing the versatility of these materials. Local piezoelectric measurements utilizing piezoresponse force microscopy confirm the expected piezoelectric and ferroelectric behavior of NBTO particles when dispersed within the chitosan matrix. This research introduces a novel class of biocompatible nanocomposite materials, combining impressive structural attributes, enhanced dielectric properties, and piezoelectric capabilities. The outcomes of this study hold substantial promise for advanced applications in opto- and piezoelectric technologies, marking a significant advancement in biologically sourced materials with multifunctional properties.

## 1. Introduction

Harnessing the abundant mechanical energy generated by vibrations, such as those caused by human movement, transport, and sound waves has become one of the concerns of both industrial and scientific communities to ensure the ecological transition and satisfy a new technological demand respecting human health and the environment [1]. This renewable and secure energy source has the potential to greatly impact our daily lives. It would enable numerous health-monitoring devices, safety equipment, sensors, alarms, lights, and other compact devices to operate autonomously, reducing our reliance on external power sources such as the electricity grid and batteries [2]. As a result, our overall energy consumption would be significantly reduced. Piezoelectric generators offer substantial potential for powering portable devices and self-sustaining electronic systems by extracting mechanical energy. The piezoelectric mechanism’s advantages over other conversion methods lie in its high energy density and scalability across a broad range of sizes.

Piezoelectric materials, renowned for their ability to convert mechanical energy into electrical signals, are considered smart materials due to their reversible nature [3]. The manifestation of this phenomenon relies heavily on the crystal lattice structure of the materials employed and the existence of a center of symmetry within their structure [3]. Poly (vinylidene fluoride) (PVDF) is widely recognized as the most prominent piezoelectric polymer employed in numerous biomedical applications, primarily attributable to its elevated piezoelectric coefficient [4]. PVDF, a polymer characterized by its crystalline structure, demonstrates remarkable piezoelectric properties [4]. While PVDF possesses a higher piezoelectric coefficient compared to other polymers, it falls short when compared to piezoceramics, which typically have a d_33_ value ranging from 13 to 28 pC/N [5]. However, PVDF is characterized by an inadequate adhesion to other materials, limited thermal stability, and the inability to generate uniform films [4].

Currently, the majority of commercially available piezoelectric generators are based on lead-containing materials. In particular, those relying on lead zirconate titanate (PZT) have demonstrated superior piezoelectric performance in numerous applications [6,7,8]. However, the utilization of lead-based materials presents significant challenges regarding environmental compatibility. Consequently, there is a pressing requirement to develop lead-free alternatives that can exhibit comparable piezoelectric properties to those achieved with lead-containing materials [9].

Lately, there has been a growing attention to utilizing biomaterials as alternatives to synthetize piezoelectric materials. Biomaterials offer superior biocompatibility, bioactivity, biodegradability, low immunogenicity, antimicrobial properties, and flexibility [3,10,11]. As a result, the scope of piezoelectric applications can be extended to the development of self-powered wearable electronic devices. Extensive research has been conducted on the piezoelectric properties of biobased materials originating from natural polymers such as cellulose, chitin, and chitosan. Chitosan, an organic polymer, with the formula (C_6_H_11_NO_4_)n is an unbranched polysaccharide composed of β-1,4-d-glucosamine, obtained through the deacetylation process of chitin [12]. Chitin is present in the exoskeletons of crustaceans and the cellular walls of mushrooms and fungi [13,14]. Incorporating biopolymers into piezoelectric applications empowers polymers to operate effectively in wearable and pliable smart devices. Nonetheless, research pertaining to the utilization of chitosan in piezoelectric applications remains relatively constrained. Some studies have explored the piezoelectric properties of commercial chitosan, and findings indicate that its piezoelectricity is comparable to that of PVDF [15,16].

Nanocellulose and chitosan have gained attention for their potential applications in various fields, including biodegradable energy harvesters and sensors. One key characteristic that makes these materials promising is their piezoelectricity, which enables them to generate electrical charges under mechanical stress [17]. In particular, as smart devices continue to proliferate within the Internet of things (IoT), concerns have arisen regarding the environmental impact associated with waste and emissions [18]. Using biodegradable piezoelectric membranes to generate energy locally offers a sustainable and economically viable solution for powering the IoT, in contrast to nondegradable materials or batteries. This approach aligns with the goal of minimizing the environmental impact while maintaining an efficient device functionality.

In this current study, we report on the synthesis of bioderived and flexible nanocomposite films by incorporating Na_0.5_Bi_0.5_TiO_3_ (NBTO) nanoparticles into a chitosan polymer matrix using a solution-casting approach. The structural, microstructural, optical, dielectric, and piezoelectric properties of these films with different NBTO contents ranging from 2 to 10% are investigated.

## 2. Materials and Methods

### 2.1. Preparation of NBTO Powder

The traditional solid-state process was used to prepare the Na_0.5_Bi_0.5_TiO_3_ (NBTO) powders. High-purity precursors including Na_2_CO_3_ (99%), Bi_2_O_3_ (99%), TiO_2_ (99.9%) were employed. The powder was measured, combined in accordance with the stoichiometric ratio, and thoroughly ground with ethanol in an agate mortar. The calcination was completed using a heating rate of 300 °C/h for 3 h at 900 °C. The powders were then reground in an agate mortar to get the final NBTO powder.

### 2.2. Preparation of Chitosan Polymer

The exoskeleton of the pink shrimp, scientifically known as “*P. longirostris*”, was utilized in the production of chitosan. It came from a Tunisian processing factory.

To eliminate organics, proteins, and other contaminants, shells were recovered from shrimp feces and cleaned under running water. The tissue was then removed from the shells by boiling them in water for 0.5 h, and then they were dried for 2 h at 100 °C to destroy the chitin’s crystal structure [19]. In the end, a blender equipped with a sieve with a 0.1 mm diameter was used to break the dry shells. The obtained raw material was sealed in bags and kept in a freezer at 4 °C.

#### 2.2.1. Demineralization

To avoid chitin’s hydrolysis, only diluted hydrochloric acid was employed to dissolve the calcium carbonate in the shells [20]. With steady stirring, the powder was demineralized with 2M HCl (1/10, *w/v*) at 60 °C for 150 min. The decalcified shells underwent filtering and neutral pH washing with distilled water.

#### 2.2.2. Deproteinization

Dried shells were demineralized under the same experimental conditions. With stirring, the dehydrated shells were deproteinized in 3 M of sodium hydroxide (NaOH) (1/10, *w/v*) at 80 °C for 120 min. The material was filtered, cleaned, and dried at the end of this procedure, as mentioned before in the demineralization method.

#### 2.2.3. Decoloration

‘‘*P. longirostris*’’ crustacean chitin was slightly pink in color. The pigment traces that give this hue its existence was eliminated using a light oxidizing procedure H_2_O_2_ (0.1 M), HCl (10 mM) in a volume ratio of 9:1 and acetone (1:5 *w/v*) for 10 min. The material was then rinsed and dried for two hours at room temperature [21].

#### 2.2.4. Deacetylation

Via the method proposed by Galed et al. [22], deacetylation was required for the process of conversion of chitin to chitosan (CS). With continual stirring at 110 °C and 15 M NaOH (1/20, *w/v*), chitin was deacetylated. After five hours, the sample was put under a hood and allowed to cool for 30 min at ambient temperature. Chitosan was subsequently rinsed with distilled water until the filtrate reached a pH of 7, then dried at 60 °C for twenty-four hours.

### 2.3. Preparation of Films

To prepare the films, the required quantity of (3 wt%) chitosan polymer was dissolved in (1 wt%) acetic acid for 3 h at room temperature.

**Figure 1:**
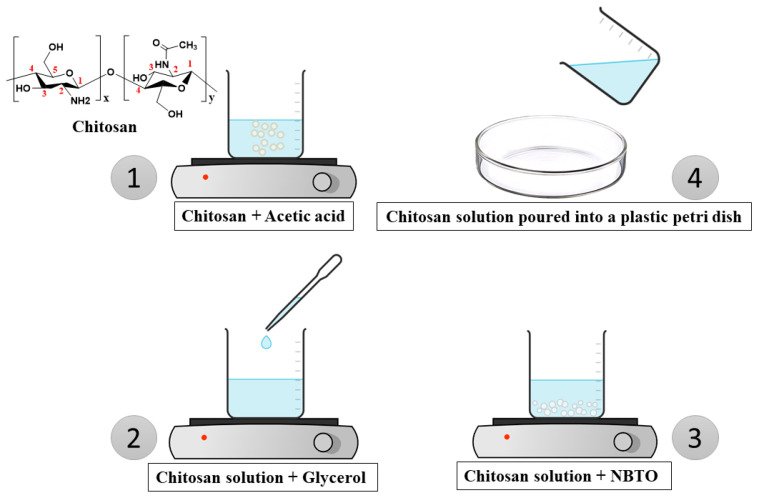
Schematic representation of the synthesis of the chitosan + NBTO membrane.

Then, while continuously stirring, (2 wt%) of glycerol was added to the solution. Following that, the NBTO powder was combined with the polymer solution using a magnetic stirrer for three hours in various mass ratios of 2%, 4%, 6%, 8%, and 10%, as shown in Figure 1.

The hybrid nanocomposite’s preparation somewhat resembled other procedures that have been employed in the past [20,23]. The solution was produced and placed in a plastic Petri dish, which was placed in an oven for drying for 12 h at 40 °C.

### 2.4. Characterizations

The NBTO powder and CS/NBTO composite films were analyzed using a Bruker Discover Advance D8 diffractometer with CuKα = 1.5406 Å for the XRD analysis. The morphology of the composites was observed using an FEI Quanta 200 FEG Environmental SEM. Infrared spectroscopy measurements of the composites were performed using an FTIR spectrometer IS50 ATR, collecting spectra within the range of 4000–400 cm^−1^. Raman spectroscopy was carried out by a Renishaw micro-Raman spectrometer, with a green laser excitation wavelength of 514.5 nm. The optical properties of the prepared systems were investigated using a Jasco V-670 UV–VIS spectrophotometer in the wavelength range of 200–800 nm at ambient temperature. As in these samples, a scattering of light occurs, the reflectance and transmittance were captured by a horizontal integrating sphere PIN757. The samples were placed in the lower part of the integrating sphere to ensure that the specular and scattered light components were captured at the same time. Dielectric investigations were conducted at room temperature using a Solartron Impedance analyzer SI-12060 over a frequency range of 100 Hz to 1 MHz. The surface morphology of the CS/NBTO10% composite was examined at the local scale using the AFM technique in alternative contact mode, also known as Tapping™ mode (TM-AFM). Then, to investigate the piezoelectric and ferroelectric properties, the dual AC resonance tracking (DART) method [24] from the PFM technique was employed under environmental conditions by using a commercial MFP-3D microscope (Asylum Research/Oxford Instruments, Santa Barbara, CA, USA). For these measurements, Pt-coated silicon tip and calibrated cantilever with a stiffness of 2.34 N·m^−1^ (PPP-EFM, Nanosensors) were utilized as the conductive probe. For PFM imaging, an AC driving voltage of 4 V was applied to the AFM tip. This allowed for the visualization and analysis of as-grown domain patterns of the sample under investigation. Additionally, PFM in spectroscopic mode was employed to record local piezoelectric hysteresis loops at the free surface (without top electrode) of the composites, giving access to the switching behavior and electromechanical activity of the probed domain beneath the tip. Such spectroscopic PFM analyses were performed by applying a continuous AC voltage (4 V) superimposed to an intermittent DC bias voltage, by selecting the remnant mode (i.e., at zero bias) in order to minimalize the electrostatic contribution while promoting the electromechanical response [25].

## 3. Results and Discussion

### 3.1. Structural Investigations

**Figure 2:**
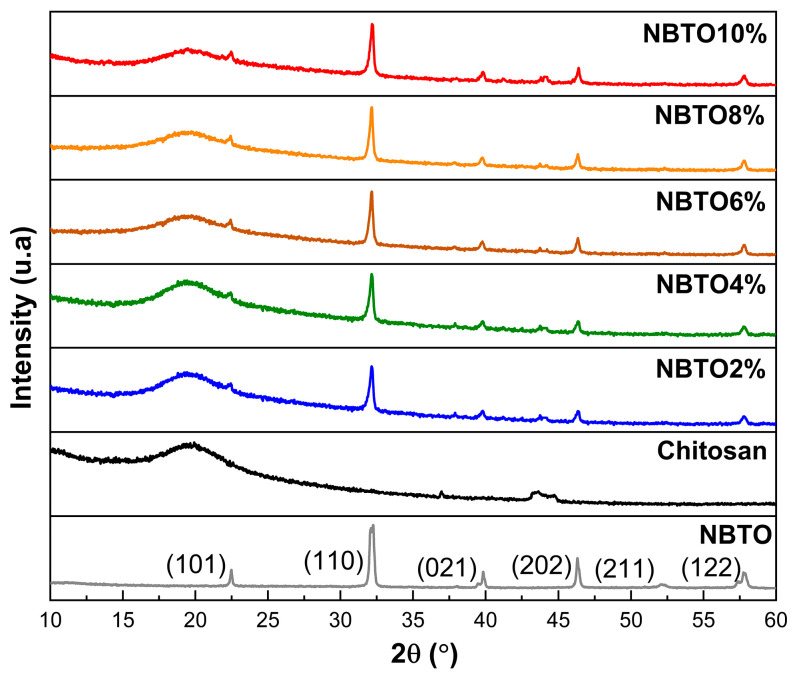
XRD of nanocomposite chitosan/NBTO.

Figure 2 illustrates the results of the X-ray diffraction patterns of the NBTO, CS, and CS/nano-NBTO composite membranes.

The NBTO powder exhibits a single perovskite phase without heterogeneous peaks, as can be shown in Figure 2. In the rhombohedral phase, the NBTO powder is indexed with the space group R3c [26]. The Debye–Scherrer equation was used to determine the NBTO crystallite size from the FWHM of the peaks which came out to 11.9 nm (1) [27]:

(1) D=k λβcos⁡θ

with *D*. the average crystallite size; *k* = 0.9, Scherrer’s constant; *λ* (CuKα) = 1.5406 Å, the wavelength of the radiation used; *β*, the peak width at half-maximum (FWHM); and *θ*, the Bragg angle of the most intense peak.

At 2*θ* = 19.6, the characteristic CS membrane peak could be seen which can be associated to the semicrystalline nature of the chitosan [28]. According to Pawlak et al. [29], chitosan’s semicrystallinity results from the compact arrangement of polymer molecules and the presence of potent intermolecular hydrogen bonding.

Other diffraction peaks appeared with the increase in the amount of NBTO added at 22.44°, 32.16°, 39.80°, 46.39°, and 57.81° of CS/nano-NBTO composite membranes. These diffraction peaks were assigned to the (101), (110), (021), (202), and (122) planes of rhombohedral NBTO [30]. The XRD spectra of composite films revealed an increase in the intensity of the principal peaks with an increasing quantity of NBTO nanoparticles. The variations in intensities are caused by variations in the amounts of NBTO nanoparticles contained [31]. Hence, the XRD results underlined the good dispersion of NBTO nanoparticles on the chitosan matrix.

### 3.2. Vibrational Investigations

**Figure 3:**
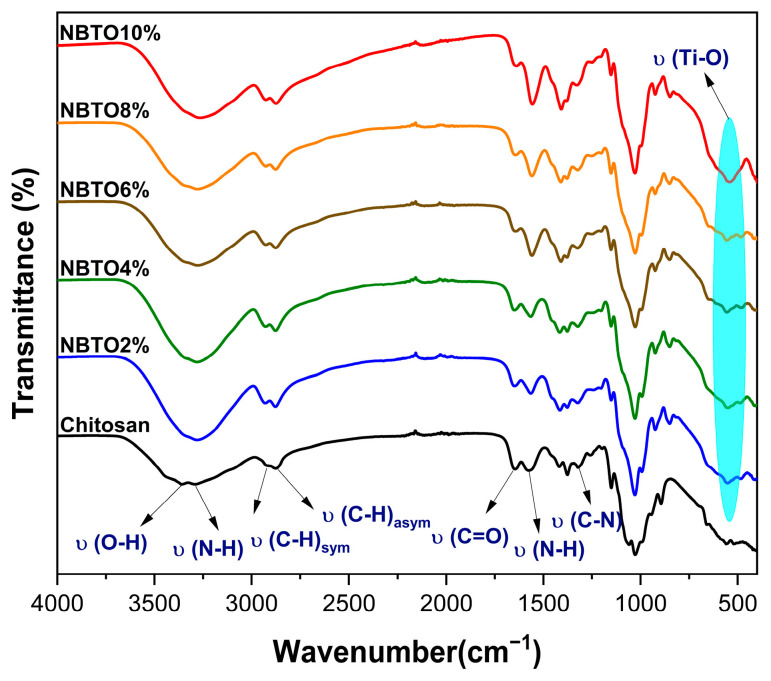
FT-IR spectra of nanocomposite chitosan/NBTO.

The infrared spectra of chitosan modified with NBTO is depicted in Figure 3. The infrared spectra of CS/NBTO, as seen in Figure 3, has a Ti-O stretching vibration peak close to 541 cm^−1^, which belongs to the NBTO characteristic peak [32]. The stretching vibrations of attached groups such as N-H and O-H on the chitosan polymer can be identified by the broadband peak of the modified chitosan’s infrared spectra at 3300 cm^−1^. The asymmetric stretching vibrations of the C-H group in the chitosan chain are represented by the band at 2864 cm^−1^, while the symmetric stretching vibrations are represented by the band at 2916 cm^−1^. These bands, which are hallmarks of polysaccharides, can be seen in various polysaccharide spectra [33,34]. Residual N-acetyl groups were identified through characteristic bands observed at 1650 cm^−1^ (corresponding to the C=O stretching of amide I) and 1317 cm^−1^ (representing the C-N stretching of amide III) [35]. Figure 3 also shows a band near 1562 cm^−1^ which resembles that of the N-H bending of the primary amine [36]. All found bands agreed rather well with what was discovered by others in previous studies [37,38].

The IR spectra present some changes as a result of the NBTO addition. The spectra clearly show a correlation between the quantity of NBTO particles present in the chitosan matrix and the width of the bands related to the N-H and O-H groups. Additionally, the appearance of the NBTO’s characteristic peak is evident as the number of NBTO particles increases. This may be a sign that coordination bonds are forming between different groups of chitosan and NBTO nanoparticles. As a result, it was determined that NBTO particles would be found between chitosan chains that were connected via functional groups, indicating that NBTO had been successfully added onto the surface of chitosan [39]. All the main FT-IR bands and vibrations are recorded in Table 1.

Furthermore, a spectrum analysis (FT-IR) was used to calculate the chitosan’s degree of deacetylation (*DDA*% = 87.9%), using the following formula as suggested in the Equation (2) [40].

(2) $$DDA\%=100-[\left(A_{1650}\;{cm}^{-1}/A_{3363}\;{cm}^{-1}\right)*\;100/1.33]$$

with: *A*_1650_ cm^−1^, the absorbance at 1650 cm^−1^ of amide I; *A*_3363_ cm^−1^, the absorbance at 3363 cm^−1^ of the hydroxyl band; and 1.33 is a correction factor used to normalize absorbance values.

**Figure 4:**
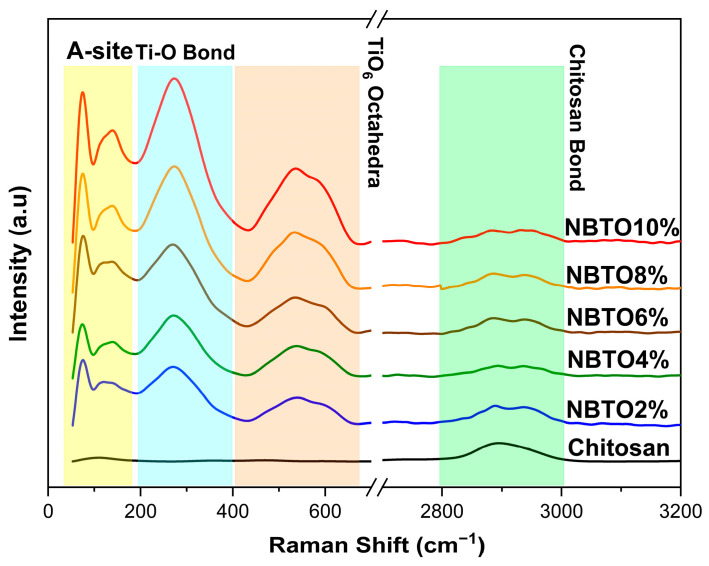
Raman of nanocomposite chitosan/NBTO.

Figure 4 shows the ambient temperature of Raman spectra of the composite samples CS/NBTO. Each of these spectra agrees well with those mentioned in the bibliography [41,42].

The spectra display the aliphatic vibrations νCH, which were attributed to the fingerprint region of chitosan that appears around 2900 cm^−1^ [43,44]. As is widely known, Raman spectroscopy is a sensitive technique for detecting structural changes and for investigating how phonon modes change with temperature, pressure, etc. As previously stated, NBTO crystallizes in the rhombohedral R3c phase, and this phase persists until approximately 200 °C [45]. The sodium bismuth titanate’s (NBTO) Raman response was shown to be similar with earlier results [46,47]. Note that the provided Raman spectra demonstrate the presence of a low-frequency mode at a wavelength of about 70 cm^−1^. Despite its obvious significance, many studies that examined NBTO’s Raman spectra did not take this last frequency component into account. However, certain authors [48,49] had a particular interest in this specific region. For example, Siny et al. examined the low-frequency mode’s behavior in order to emphasize the presence of a central component in NBTO single crystals that is well known in relaxor ferroelectrics [49].

Other research studies on ceramic systems [50] and NBTO-xBT single crystal have examined the temperature variation in the Raman spectra of the low-frequency mode for studying the BaTiO_3_ doping effect in an NBTO system. As shown in Figure 4, the Raman spectrum of NBTO consists of three Raman regions: two distinctive bands with a frequency mode around 70 cm^−1^ and 150 cm^−1^ that belong to the vibrations of Bi-O and Na-O, respectively, which define region 1. Furthermore, the intense vibrational Raman mode around 240 cm^−1^ that mostly involves B-site cations, thus Ti-O vibrations, which was revealed to be the main feature in our spectra [46]. According to Maria et al. [51], the last region modes between 400 cm^−1^ and 700 cm^−1^ are BO_6_ tilting modes, which means they are a consequence of TiO_6_ octahedral vibrations. These modes, which include oxygen atom variations in perovskites while cations are essentially at rest, are widely known [52].

In the current investigation, there was no discernible frequency variation when NBTO was added to the CS polymer. However, it was found that as NBTO concentrations increased, the intensity ratio of NBTO modes also increased.

### 3.3. Microstructural Analysis

**Figure 5:**
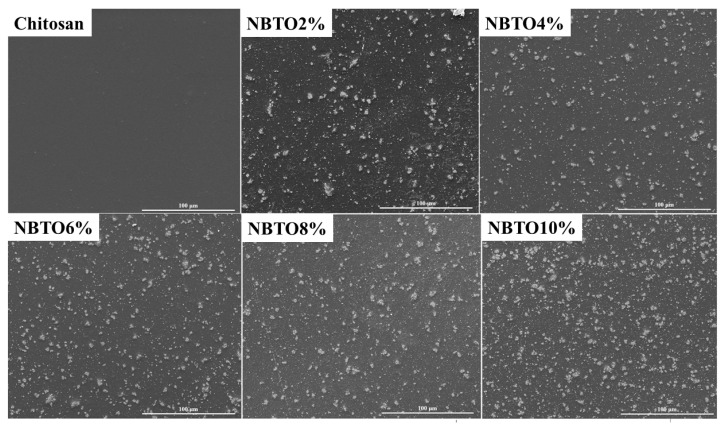
SEM images of nanocomposite chitosan/NBTO.

We carried out SEM studies on a variety of CS/NBTO composites with various doping concentrations, as shown in Figure 5, in order to more clearly see the distribution of NBTO nanoparticles in the CS matrix. Two zones can be seen in Figure 5, the first one is dark and the other is light. Indeed, the light particles are the nanoparticles, while the black region is the CS matrix. On the other hand, Figure 5 illustrates how the NBTO nanoparticles are arbitrarily overlaid and interlocked with other particles to form linked grains with a lot of interspaces. The pure CS film also demonstrates the absence of pores and cracks, as previously described in the literature [53,54]. This shows that the solution-casting technique used can successfully disperse the NBTO filler in the CS matrix. The packing of the particles became denser as the NBTO content increased, demonstrating the remarkable interaction between the CS polymer and NBTO particles.

### 3.4. Dielectric Studies

The potential of chitosan nanocomposite films in terms of their dielectric property remains largely untapped. The dielectric property of CS composite materials possesses great technological promise due to its biocompatibility, cost-effectiveness, and processability. These materials have the potential for various applications in next-generation actuators, fuel cells, sensors, self-regulating heaters, and capacitors. Moreover, by tuning the dielectric property of flexible and biocompatible films, they could serve as artificial muscles for future medical treatments and play a significant role in the development of “smart skins” in the medical field [55,56,57]. Limited research has been conducted on the dielectric properties of specific synthetic polymer composites [58].

**Figure 6:**
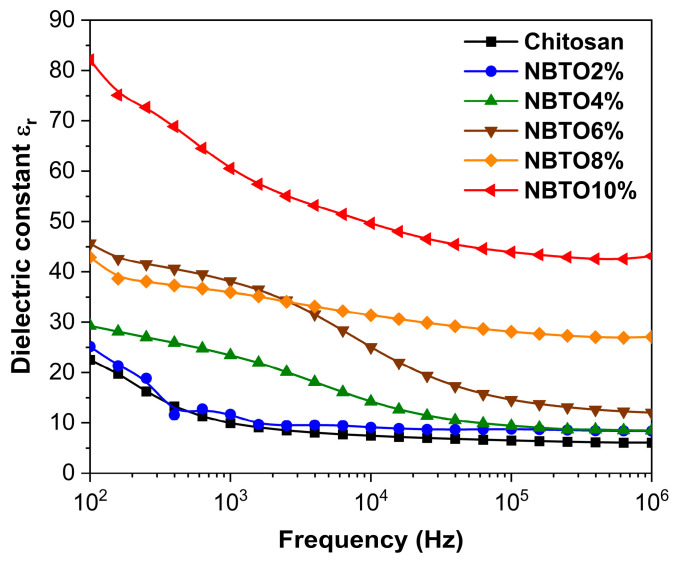
Variation of dielectric constant of chitosan/NBTO films with frequency.

Figure 6 illustrates the measured dielectric constants of the chitosan composite films at room temperature, as a function of the NBTO content, across a frequency range spanning from 100 Hz to 10^6^ Hz. All the composite films displayed higher dielectric values when compared to the pure chitosan film (as illustrated in Figure 6). The increase in the quantity of NBTO particles resulted in an elevation of dielectric values due to the corresponding increase in space polarization within the interface of the composite materials. It is a commonly observed trend for dielectric substances to exhibit a gradual decrease in their dielectric constant as the frequency of the electric field rises. This phenomenon is attributed to the inability of the polarization mechanism to keep up with the rapid changes in the electric field at higher frequencies which could be attributed to the effect of the electrode contacts. Consequently, the contribution of polarization to the overall dielectric constant diminishes at high frequency due to dielectric relaxation [23]. Thus, the dielectric permittivity values of the samples should be taken at low frequency for comparison.

For the NBTO powder fractions of 2%, 4%, 6%, 8%, and 10%, the dielectric constant values showed an increase compared to the dielectric constant of the pure polymer, starting from 22 and rising to 26, 29, 43, 46, and 82, respectively, at 100 Hz. This increase in relative permittivity can be attributed to two main factors: firstly, it is due to the higher concentration of NBTO powder in the composite. When subjected to an external electric field, a significant accumulation of charge occurs, leading to a polarization at the interface between the particles and the matrix. This accumulation of charge and subsequent polarization contribute to the overall increase in the dielectric constant [32]. Secondly, the inherent dielectric constant of the NBTO powder itself is approximately 600, which is considerably higher than that of the CS polymer. This significant disparity in dielectric constants between the NBTO powder and the CS polymer further contributes to the overall increase in the dielectric constant of the CS/NBTO composite with increasing filler doping concentrations.

### 3.5. Local Piezoelectric Responses

**Figure 7:**
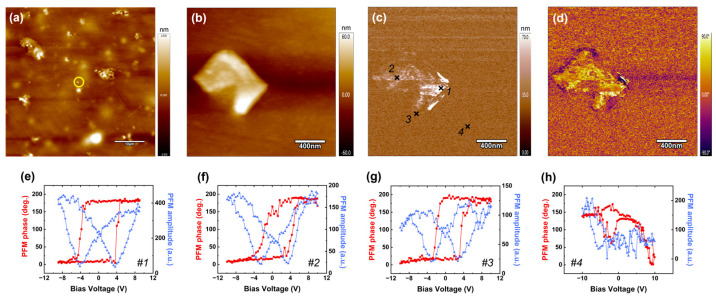
(**a**,**b**) AFM topographies and PFM (**c**) amplitude and (**d**) phase images recorded on the CS/NBTO10% film. (**e**–**h**) Remnant phase and amplitude piezoloops measured at points 1 to 4 in (**c**).

The surface morphology and piezoelectric response of the CS/NBTO10% NCs were investigated at the nanoscale by TM-AFM and PFM, respectively. The typical AFM topography recorded over a large region (50 × 50 µm^2^) is shown in Figure 7a, where a relatively even distribution and variable grain size of NBTO particles in the CS/NBTO10% thin film is observed. Such a rough surface is in agreement with the hybrid polymer/ceramic nature of the sample. Figure 7b presents a zoomed-in image of the yellow-circled area on the previous image. It clearly shows a rectangular object of nanometric size, which can be thoroughly attributed to an NBTO particle.

Figure 7c,d exhibit the as-grown PFM amplitude and phase signals acquired over the region presented in Figure 7b. The contrasts observed in both amplitude and phase images strongly suggest the presence of piezoelectric and ferroelectric activity associated to a well-defined domain structure within the CS/NBTO10% film. In addition, this activity is confined to the area previously assigned to the nanoparticle, while no piezoresponse seems to be detected beyond this region. These results reveal the expected distribution of polarized ferroelectric domains into the NBTO particles and suggest the polymer matrix has no electroactive properties. Due to the elaboration method of the composite films, we cannot exclude the presence of a superficial polymer layer covering the ceramic particles. Although this thin chitosan layer over the surface of the nanofillers reduces the quality of the surface morphology recorded by TM-AFM, it does not prevent the detection of local spontaneous piezoactivity when using the PFM technique. Such result was previously reported in PVDF/BaTiO_3_ composite films [25] and explained by the very high electric field produced beneath the tip combined with the DART-PFM method, which amplifies the electromechanical response.

To better assess the piezoelectric behavior of the NBTO particles as well as the chitosan matrix, we further probed the region depicted in the image of Figure 7b–d using the spectroscopic mode of PFM. More specifically, four specific locations were investigated, numbered from 1 to 4 on Figure 7c. The characteristic measured piezoloops at each location are shown in Figure 7e–h. First, when probing areas corresponding to the previously identified NBTO particle (points 1 to 3 in Figure 7c), well-defined amplitude and phase (simultaneously recorded) remnant PFM loops are obtained. Indeed, a clear hysteresis associated to the 180° phase difference signifying two constant states with opposite polarity for phase loops and a butterfly-shaped signal for amplitude loops are observed, which are both the signature of nanoscale ferro- and piezoelectricity. In addition, such phase PFM responses provide evidence of the efficiency of domain polarization reversal into the NBTO particles. On the other hand, no piezoresponse was detected when probing the regions surrounding the particle and associated with the polymer matrix, as observed in Figure 7h (point 4). These results corroborate the as-grown PFM activity observed by scanning the CS/NBTO10% film surface (Figure 7c,d) and clearly confirm the electromechanical behavior of the NBTO inclusions and the nonpolar nature of the chitosan matrix. In addition, the low coercive voltage determined from the phase loops (about 3.9 V) is in full agreement with the inorganic nature of the probed compound, where the crystalline structure is more homogeneous compared to organic ones. Now, by carefully examining point 3, we observe that the probed area is slightly outside of the particle. Given that the matrix is non-piezoelectric, the detected response is attributed to the particle, which is buried deeper beneath the polymer layer. It is further supported by the lower PFM amplitude detected on the corresponding loop compared to the two other piezoloops presented in Figure 7e,f. This result further demonstrates the high sensitivity of the PFM tool for probing the electromechanical behavior of nanoparticles dispersed within a low-permittivity polymer matrix.

### 3.6. Optical Investigations

**Figure 8:**
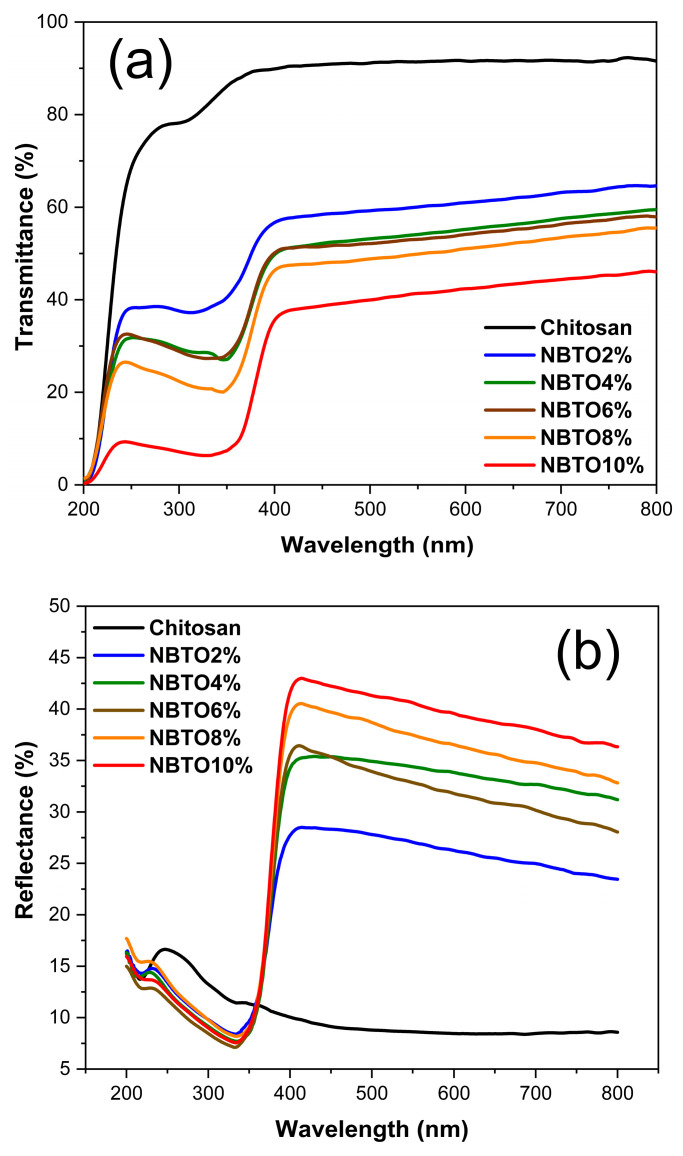
Optical response of pure chitosan and nanocomposites (**a**): transmittance (**b**) reflectance.

Figure 8a,b show the total transmittance (T) and reflectance spectrum (R), respectively, for different samples. The chitosan sample exhibits the typical behavior which is expected for this material with a thickness of around 30 µm. The refractive index is slightly above 1.5 between 800 nm and 400 nm, and the absorption coefficient strongly increases below 250 nm. This is consistent with literature data [59].

The spectra of the composites exhibit a sharp drop around 370 nm, which corresponds to the signature of the NBTO band gap described in literature [60]. However, one also observes another outcome related to the introduction of NBTO in the chitosan matrix, which is surprising. In fact, even at low NBTO concentrations, a strong drop of the transmittance and a high increase in the reflectance occurs in the 400–800 nm range compared with the pure chitosan sample. For instance, the reflectance of the NBTO2% composite increases from about 7% to more than 25%.

It is well known that the optical properties of composites, displaying nonuniformity at a scale much smaller than the light wavelength., can often be described by an effective dielectric constant framework of effective medium approximations (EMA) [61]. However, for our composites this approach failed. The observed behavior of the spectra could not be described by usual mixtures of the dielectric functions of the two phases. It turned out to be impossible to fit the composite spectra using the main effective medium models (Bruggeman, Maxwell Garnett, Looyenga) [61] or even a more general EMA approach such as the Bergman representation [62].

The shapes of the experimental reflectance spectra, with a continuous increase in R with energy in the 400–800 nm range, actually suggest a scattering mechanism. In order to explore this idea, we modelized the spectra with a Mie theory approach using the commercial spectral ray-tracing simulations software SPRAY 2.58 (https://www.wtheiss.com) [63]. In this model the nanocomposite was described by a matrix composed of chitosan and embedded NBTO particles as spheric Mie scatterer. Moreover, an air–composite interface was introduced in order to better simulate the experimental conditions of the reflectance measurement of the samples. The only adjustable fit parameters were the size distribution and the volume fraction of the NBTO particles. For the NBTO particles, the dielectric function in the range of 300–800 nm was taken from [60] as it matched well the observed reflectance spectra of our pure NBTO powders, and for the chitosan matrix, the dielectric functions extracted from the R and T spectra of the pure chitosan sample were taken.

**Figure 9:**
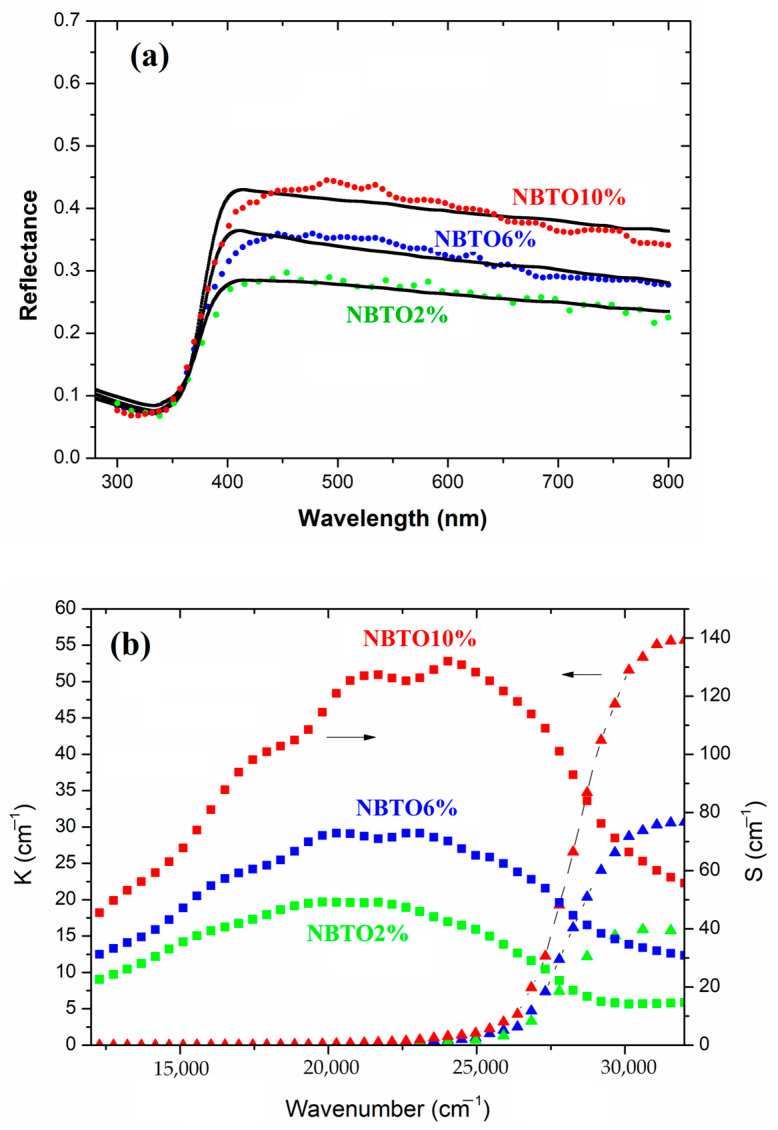
NBTO as Mie scattering particles embedded in the chitosan matrix. (**a**) Examples of fits for 2%, 6%, and NBTO10%: solid lines are experimental spectra, and dotted curves are modelized spectra using Mie scattering approach. (**b**) Corresponding Mie scattering values K (▲) and S (■).

The simulation consisted of tracing the rays of 15,000 photons impacting the composite surface for each wavelength with a maximum of 10,000 possible scattering events per photon in order to model the scattering and absorption behavior of the composite. The scattering and absorbing medium is characterized by the following quantities: S = scattering probability/distance and K = absorption probability/distance. Figure 9a exhibits the respective reflectance curves of the 2%, 6%, and 10% samples, and in Figure 9b, the corresponding K and S curves are shown. The slight fluctuations of the fit curves could be smoothed by using a much higher number of photons, but this makes calculations very time-consuming without improving the main outcome.

As illustrated in Figure 9a the Mie model can fairly reproduce the optical response in the 450–800 nm range with a very limit number of fit parameters. It turns out that only particles with diameter sizes between 100 nm and 400 nm significantly contribute to the scattering behavior. This explains why the determined volume fraction of the scattering particles in the model is quite low: between 0.1% (NBTO2%) and 0.4% (NBTO10%). Bigger particles or even clusters as revealed by SEM (Figure 5) might only slightly contribute to the higher reflectance by an effective medium mixture of the two phases. The spectral region around 370–400 nm is dominated by the NBTO bandgap and is illustrated by the sharp increase in K in Figure 9b. Note that 27,000 cm^−1^ corresponds to 370 nm. However, a more detailed study of the simulation reveals that small particles with 100 to 150 nm size also contribute to the shape of the absorption edge around 400 nm. Increasing the weight of these particles in the distribution at the cost of those of 200–400 nm size leads to a bitter fit of the edge but deteriorates the fit in the red region of the spectra (above 600 nm). The examples shown in Figure 9a are the result of the best compromise in the size distribution.

Additionally, the Tauc relation (3) [64] can be used to determine the direct/indirect optical bandgap (*Eg* _dir._/*Eg* _ind._) of all composites (CS/NBTO):

(3) $${\left(\alpha h\nu \right)}^{n}=k(h\nu -Eg)$$

where *hν* is the photon energy, h is Plank’s constant, α is the absorption coefficient, *Eg* is the optical energy gap, *k* is a constant, and for direct transitions, *n* = 2, while for indirect transitions, *n* = 1/2. The optical bandgap value, *Eg*, is obtained by intersecting this straight line with the x-axis on a graph of (*αhν*)*^n^* against *hν* [65,66].

**Figure 10:**
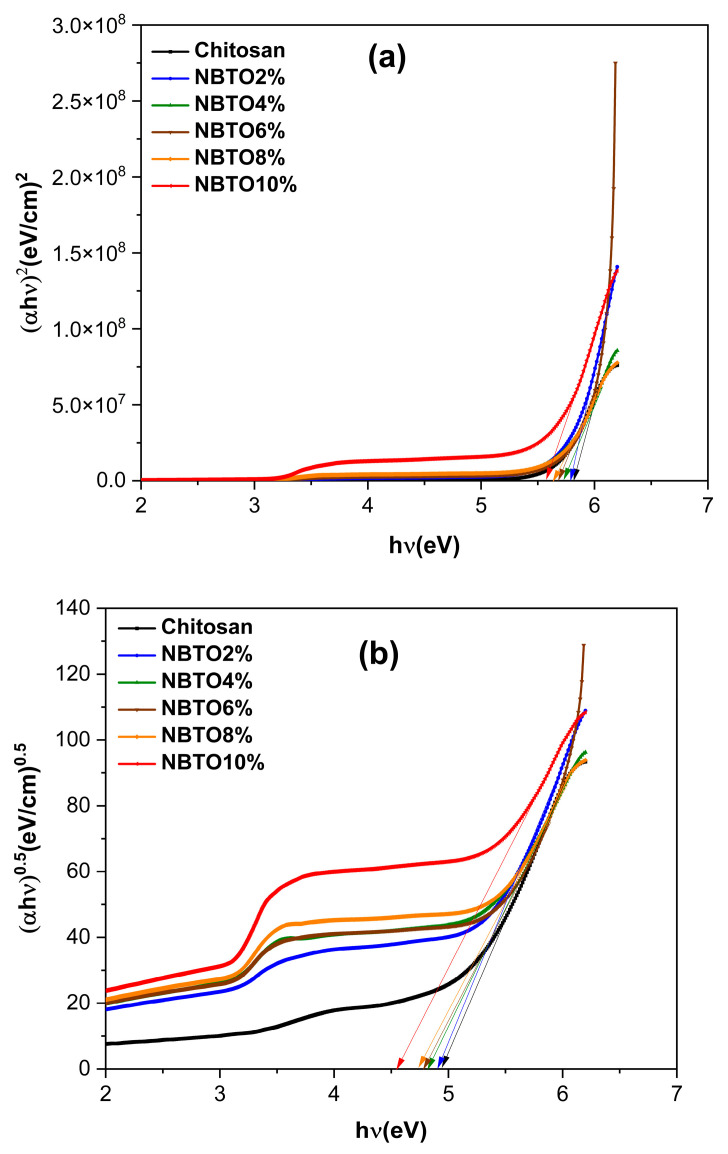
Optical bandgap plots’ (**a**) direct and (**b**) indirect status of CS/NBTO films.

Figure 10a,b show the variations of the direct/indirect optical bandgap energies of different thin films. The graph of (*αhv*)*^2^* as a function of *hv* of chitosan gives the energy bandgap, with an *Eg* value of 5.81 eV, while for the indirect optical bandgap, *Eg* is near to 4.94 eV, which agrees with other previous works [64]. The variation of optical bandgap energies of thin films is related to the concentrations of NBTO. It is clear that the increase in the quantity of NBTO led to a reduction in *Eg*; for example, for CS/NBTO2%, the *Eg* value is 4.90, while for CS/NBTO10%, *Eg* is around 4.55 for indirect transitions. These results are consistent with those that have been published [65,67,68]. The deduced *Eg* values of the CS/NBTO films are recorded in Table 2.

## 4. Conclusions

In summary, the successful fabrication of flexible composite films combining polymer and ceramic was achieved by incorporating sodium bismuth titanate (NBTO), a lead-free piezoelectric material, into a chitosan (CS) polymer matrix derived from the shells of *Parapenaeus longirostris* shrimp. These films were synthesized with varying amounts of NBTO filler and subjected to thorough investigations. The produced chitosan membrane and the nanocomposite chitosan/NBTO membrane were characterized by diverse techniques. X-ray diffraction (XRD) analyses validated the presence of characteristic peaks corresponding to both chitosan and NBTO, which exhibited a pure perovskite phase with the R3c space group. The Fourier transform infrared (FT-IR) spectra of CS/NBTO films indicated an increasing prominence of NBTO’s distinctive peak with higher NBTO particle concentrations, potentially indicating the formation of coordination bonds between different chitosan groups and NBTO nanoparticles. Scanning electron microscopy (SEM) images depicted two discernible regions: a dark region representing the CS matrix and a lighter area signifying the NBTO nanoparticles’ presence. These nanoparticles were observed to arrange arbitrarily, interlocking with other particles to create interconnected grains interspersed with ample gaps. The optical behavior above 400 nm was largely dominated by a light scattering of NBTO nanoparticles within the diameter range of 100–400 nm, while the NBTO bandgap introduced a sharp edge around 370 nm. Incorporating NBTO particles into the polymer matrix notably enhanced the dielectric permittivity, surpassing values reported in previous studies. Further, the variations of the direct/indirect optical bandgap energies were determined, and it was found that the increase in the quantity of NBTO led to a reduction in these energies. Additionally, nanoscale piezoelectric measurements conducted on the CS/NBTO10% film showcased an electromechanical behavior. This promising result paves the way for potential applications of such CS-based composites in actuator, sensor, or energy-harvesting systems.

## Figures and Tables

**Table 1:** FT-IR bands of CS/NBTO nanocomposite films.

Wave Number (cm^−1^)	Band Vibration
3361	O–H stretching
3286	N–H stretching
2916	C–H symmetric
2864	C–H asymmetric stretching
1650	C=O stretching
1562	N–H bending
1317	C–N stretching
541	Ti–O stretching

**Table 2:** Eg of CS/NBTO nanocomposite films.

Nanocomposites	*Eg*_dir._ (eV)	*Eg*_indir._ (eV)
Chitosan (CS)	5.81	4.94
CS/NBTO2%	5.78	4.90
CS/NBTO4%	5.75	4.82
CS/NBTO6%	5.68	4.78
CS/NBTO8%	5.65	4.74
CS/NBTO10%	5.57	4.55

## Data Availability

Not applicable.
